# Draft genome sequence of *Mannheimia haemolytica* strain Oliden, isolated from a calf lung infection in Argentina

**DOI:** 10.1128/mra.00997-23

**Published:** 2023-12-01

**Authors:** Agustín Franchi, Luis Fazzio, Julieta Fernandez, Federico Sisti

**Affiliations:** 1 Departamento de Ciencias Biológicas, Facultad de Ciencias Exactas, Instituto de Biotecnología y Biología Molecular CCT CONICET, UNLP, La Plata, Argentina; 2 LAPEVET-Laboratorio de Patología Especial Veterinaria, Facultad de Ciencias Veterinarias, UNLP, La Plata, Argentina; Rochester Institute of Technology, Rochester, New York, USA

**Keywords:** *Mannheimia haemolytica*, bovine respiratory disease

## Abstract

We present the draft genome sequence of a *Mannheimia haemolytica* strain isolated from a postmortem lung lesion from a calf diagnosed with bovine respiratory disease. The genome sequence was 2,749,707-bp long with 2,909 putative protein-encoding genes.

## ANNOUNCEMENT

Bovine respiratory disease (BRD) is one of the most important respiratory diseases in cattle and sheep. *Mannheimia haemolytica*, among other pathogens, contributes to the BRD. Here, we report a *M. haemolytica* sequence from a strain isolated from a feedlot cattle facility (capacity for 10,000 animals) in Oliden City, Buenos Aires Province, Argentina (35.1838°S, 57.9487°W). Sample was collected postmortem from the lung of an Aberdeen Angus heifer (10 months, 160 kg). The animal showed no previous clinical signs and had not received any antibiotic treatment. At necropsy, the lungs showed grade 2 autolysis (on a scale of 0–3), and the extent of lung lesions was quantified as 64% according to the classification proposed by Fajt et al. (1). The morphological diagnosis attributed the cause of death to severe fibrinous bronchopneumonia. Microscopic analysis revealed the presence of fibrin in the alveolar and interlobular septa, hemorrhages, necrotic leukocytes (oat cells), thrombosis, vasculitis, alveolar edema, and the presence of macrophages in the alveolar lumen. Additionally, epithelial necrosis was observed in the bronchi. Furthermore, fibrinous pericarditis was identified as a concurrent pathological finding.

Ancillary tests were conducted for bovine respiratory syncytial virus (BRSV), bovine viral diarrhea virus, bovine parainfluenza virus type 3 (BPIV3), and bovine herpes virus-1. Immunohistochemistry confirmed the presence of BPIV3 in lung samples, while PCR detected the presence of BRSV.

Bacteria were isolated from the lesions present in the animal’s lungs, cultured in brain heart infusion (BHI) agar, and incubated for 24 hours at 37° C. Identification was performed by standard classical microbiological techniques and confirmed by PCR as described previously (2).

A single colony was selected and grown in liquid BHI, for 18 hours at 37° C, 200 rpm. DNA was extracted using a commercial kit (catalog number 69504; Qiagen), following the manufacturer’s protocol for Gram-negative organisms, and quantified by Nanodrop. The genomic DNA was neither sheared nor size selected.

Library preparation was performed using Illumina Nextera chemistry, with sequencing on the Illumina NextSeq 550 platform (2 × 150 bp reads). Quality control was performed by fastQC v 0.12.0 (http://www.bioinformatics.babraham.ac.uk/projects/fastqc). Sequencing produced 17,169,496 pairs of reads. The raw data were trimmed at the Bacterial and Viral Bioinformatics Resource Center web server selecting SPAdes as the assembly strategy while all other settings remained at their default values (3). Assembly generated 75 contigs (>2,000 bp). The largest contig was 155,253 bp and the smallest was 2,007 bp. The N50 parameter for the contigs was estimated to be 88,566-bp long. The size of the assembled *M. haemolytica* Oliden strain was 2,749,707 bp with a G-C content of 41%. Genome annotation was conducted using NCBI Prokaryotic Genome Annotation Pipeline v6.4 (4). Default parameters were used for all software unless otherwise specified.

*M. haemolytica* identification was confirmed by Type (Strain) Genome Server and according to Klima et al., the *M. haemolytica* A isolate is serotype 6 (5, 6).

## Data Availability

This Whole Genome Shotgun project has been deposited at DDBJ/ENA/GenBank under accession no. JAPZQI000000000. The version described in this paper is version JAPZQI010000000. Raw reads were uploaded to the SRA database under accession no. SRX21931104.
